# EDTA enhances cementum-like tissue formation, TGF-β1 & VEGF in rat molars during regenerative endodontics

**DOI:** 10.1590/1807-3107bor-2025.vol39.068

**Published:** 2025-07-07

**Authors:** Alexandre Henrique dos REIS-PRADO, Isabela Joane Prado SILVA, Juliana GOTO, Nathália Evelyn da Silva MACHADO, Gleide Fernandes de AVELAR, Juliano Douglas Silva ALBERGARIA, Raphael Escorsim SZAWKA, Marco Cícero BOTTINO, Luciano Tavares Angelo CINTRA, Edilson ERVOLINO, Antônio Paulino RIBEIRO-SOBRINHO, Francine BENETTI

**Affiliations:** (a) Universidade Federal de Minas Gerais – UFMG, School of Dentistry, Department of Restorative Dentistry, Belo Horizonte, MG, Brazil.; (b) Universidade Estadual Paulista – Unesp, School of Dentistry, Department of Restorative Dentistry, Araçatuba, SP, Brazil.; (c) Universidade Federal de Minas Gerais – UFMG, Institute of Biological Science, Department of Morphology Belo Horizonte, MG, Brazil.; (d) Universidade Federal de Minas Gerais – UFMG, Institute of Biological Science, Department of Physiology and Biophysics, Belo Horizonte, MG, Brazil.; (e) University of Michigan School of Dentistry, Department of Cariology, Restorative Sciences and Endodontics, Ann Arbor, MI, USA.; (f) Universidade Estadual Paulista – Unesp, School of Dentistry, Department of Basic Science, Araçatuba, SP, Brazil.

**Keywords:** Edetic Acid, Fibroblast Growth Factor 2, Regenerative Endodontics, Transforming Growth Factor beta, Vascular Endothelial Growth Factor A

## Abstract

This study investigated the influence of ethylenediaminetetraacetic acid (EDTA) irrigation on cementum-like tissue formation and TGF-β1, FGF-2, and VEGF immunolabeling during regenerative endodontic procedures (REPs) in immature rat molars. The lower first molars of 12 four-week-old male rats (80 g) underwent pulpectomy in the mesial canal and were randomly categorized into two experimental groups (n = 6): sodium hypochlorite (NaOCl) – irrigated for 5 min with 2.5% NaOCl; and NaOCl-EDTA – irrigated with 2.5% NaOCl, followed by 5 min of 17% EDTA. After inducing bleeding with a size 10 K-file, the cavities were sealed. Untreated molars served as control-15d (n = 3) and immediate control (n = 3). Either immediately or after 15 days, the animals were euthanized, and the teeth were collected for histomorphometric and immunohistochemical (TGF-β1, FGF-2, and VEGF) analysis. The results were analyzed by the Mann-Whitney U-test (p < 0.05). Histomorphometric analysis revealed increased cementum-like tissue formation in the NaOCl-EDTA group compared with that in the NaOCl group (p < 0.05). Regarding growth factor immunolabeling, the NaOCl-EDTA group exhibited enhanced TGF-β1 and VEGF immunolabeling in the root tip area and the center region of the apical third of the pulp tissue, compared with that in the NaOCl group (p < 0.05); however, no significant difference was observed in FGF-2 (p > 0.05). In conclusion, the use of EDTA in REPs positively affected the formation of cementum-like tissue and TGF-β1 and VEGF in the apical region but did not influence FGF-2.

## Introduction

The regenerative endodontic procedures (REPs) aim to replace damaged structures of the dentin–pulp complex,^1,2^ promoting root development and achieving apical closure.^3^ This procedure employs principles of tissue engineering, utilizing 3-dimensional scaffold, stem cells, and growth factors, following dentin demineralization^1,4,5^and ensuring an appropriately disinfected root canal space.^3,6,7^ The dentin matrix is recognized as a reservoir of growth factors, which are pivotal for the recruitment, proliferation, and differentiation of stem cells.^3,8, 9^ Among these factors, transforming growth factor (TGF)-β1, fibroblast growth factor (FGF)-2, and vascular endothelial growth factor (VEGF) play essentials roles in the regeneration of the pulp-dentin complex.^10^

TGF-β1 acts as a chemoattractant and stimulates the activation and migration of stem cells,^8^ upregulating odontoblastic differentiation.^3,11^ This molecule also contributes to reparative dentinogenesis^3,11^and exerts potent immunosuppressive effects against pro-inflammatory cytokines.^2^ Furthermore, FGF-2 has demonstrated the potential to differentiate human dental pulp cells (HDPCs) into odontoblast lineages^12^ and stimulate angiogenesis, serving as a mitogen for pulp progenitor cells^10^ and promoting mineralized tissue production.^10^Similarly, VEGF has an angiogenic potential, fostering the differentiation of stem cells into endothelial cells.^13^ VEGF can be secreted from conditioned dentin matrix or introduced into the root canal from periapical blood.^14^ These growth factors can trigger cellular responses even at low concentrations.^15^

Regarding the irrigation protocol, the European Society of Endodontology considers irrigation with 1.5–3% sodium hypochlorite (NaOCl) to be appropriate due to its effective disinfection and tissue-preserving capabilities.^16^ In addition, the selected irrigating solution can have a profound effect on the release of growth factors.^3^ Current guidelines^16,17^advocate the use of 17% ethylenediaminetetraacetic acid (EDTA) following NaOCl irrigation in REPs to enhance the release of growth factors from dentin, thereby promoting the proliferation and differentiation of undifferentiated stem cells within the root canal space.^18^ Although other chelator/acids have been explored recently,^3,19,20^ EDTA has displayed encouraging outcomes in recent in vivo studies on animal teeth subjected to REPs, such as improved newly formed intracanal connective tissue and increased root dentin thickness.^7,20^ However, a recent systematic review suggests that the effect of EDTA conditioning on growth factor liberation remains controversial.^14^ Some investigations showed no discernible impact of EDTA on TGF-β1 release,^11,19^while others identified an increased amount of this factor.^2,8,13,21,22^

Most investigations involve laboratory studies using conditioned dentin powder or dentin discs,^3,4,8,9,11,13,19,21^ which do not fully replicate the clinical scenario of REP. Rat molars exhibit significant physiological and anatomical similarities to human teeth.^7^Furthermore, research using an animal model can be valuable for accessing the amount of newly formed mineralized tissue and the release of bioactive molecules from conditioned dentin after REP, aligning with the recommendations of clinical guidelines.^16,17^ Thus, this study aimed to investigate the influence of EDTA irrigation on cementum-like tissue formation and on the immunolabeling of TGF-β1, FGF-2, and VEGF in immature rat molars undergoing REP. The null hypothesis was that there is no difference in mineralized tissue formation and immunolabeling of growth factors between the evaluated irrigation protocols.

## Methods

### Animals

Fifteen four-week-old male Wistar rats (80 g) were used. The sample size was calculated based on data of periapical inflammation from rat molars subjected to REP in two experimental groups in a previous study.^23^Considering 95% power and an alpha-type error level of 0.05, the sample size consisted of 5 molars per experimental group. Taking into consideration possible animal deaths, one more animal was added to each group, resulting in 6 rats per group.^2,23^ The animals were housed in a temperature-controlled environment (22°C ± 1°C, 70% humidity, 12-h light-dark cycle) with ad libitum access to water and feed. This study was approved by the local Ethics Committee (CEUA 81/2020) and conducted according to the Guide for the Care and Use of Laboratory Animals of the National Institutes of Health (Bethesda, USA).

### Surgical procedure

The study protocol has been previously described.^7^ Briefly, after anesthesia was administered via intraperitoneal injection of 10% ketamine (80 mg/Kg; Ketamina Agener 10%, União Química Farmacêutica Nacional S/A, Embu-Guaçu, Brazil) and 2% xylazine (15 mg/Kg; Xilazin, Syntec do Brasil LTDA, Cotia, Brazil), the lower left or right first molar of 12 animals was isolated using special dental clamps and gingival barrier, preventing any contact with saliva.^7^ Coronal access to the mesial root canal was then carefully achieved using a sterile long neck (LN) round bur (diameter of 0.06 mm; Dentsply Maillefer, Tulsa, USA), under constant irrigation with saline solution, aided by an operating microscope (24× magnification; Alliance, São Paulo, Brazil).^2,7^ After exposing the dental pulp tissue, a pulpectomy was subsequently performed using size 10 K-files in a counterclockwise push-and-pull motion to a working length of 4 mm.^7^ The canals were then irrigated with a sterile saline solution and dried using sterile paper points.

Thereafter, the molars subjected to the aforementioned pulpectomy were randomly distributed into two experimental groups (n = 6) defined by lottery: in the NaOCl group, the mesial canals were irrigated with 2.5% NaOCl at a rate of 0.1 mL/min for 5 min; in the NaOCl-EDTA group, the mesial canals were irrigated with 2.5% NaOCl, followed by 17% EDTA, each at a rate of 0.1 mL/min for 5 min. Then, the canals were then rinsed with saline solution and dried with sterile absorbent paper points. A sterile 10 K-file was inserted 0.5 mm beyond the apex^7^ to induce bleeding and the formation of blood clot inside the mesial canal. The teeth were then sealed with PBS CIMMO HP (CIMMO, Pouso Alegre, Brazil), a mineral-oxide based cement, followed by the application of resin-modified glass ionomer cement that was light-cured (GC America Inc., Alsip, USA).

Three untreated lower left or right first molars of rats from the experimental groups (NaOCl and NaOCl-EDTA) were randomly selected as control (Control-15d, n = 3). Additionally, the lower left or right immature molars of three other animals did not receive any intervention, and they were used as Control-immediate (n = 3). These controls were included for histologic reference and observation of physiologic root development.

### Sample preparation and histological analysis

At the 4-week period (Control-immediate group) or at 15 days after REP, the rats were euthanized with an overdose of the anesthetic solution, and the hemi-mandibles were separated, dissected, and fixed (4% buffered formaldehyde; 24 h). The specimens were decalcified (10% EDTA; 45 days), dehydrated, clarified, and embedded in paraffin. Serial histological sections (5 µm) were selected from the point where the mesial root of the first molar was at its full longitudinal extension. The two first slides obtained with histological sections were selected for hematoxylin-eosin (H&E) staining, and each of the next two for immunohistochemical analysis.

All analyses of the histological sections were performed under light microscopy (400× magnification; DM4000 B; Leica Microsystems GmbH, Wetzlar, Germany) by a single experienced and calibrated operator blinded to the experimental groups. Sections stained with H&E (one section for each specimen) were used for histomorphometric analysis, and the area of the newly formed cementum-like tissue in the apical area was measured by image processing software (Leica QWin V3, Leica Microsystems, Wetzlar, Germany) and obtained in µm^2^.^24-26^

### Immunohistochemical analyses

Immunohistochemical assessments using indirect immunoperoxidase technique^27,28^for TGF-β1, FGF-2, and VEGF were performed. Antigen retrieval was achieved by immersing the histological slides in buffer citrate solution (Spring Bioscience Corporation, Pleasanton, USA) in a pressurized chamber (Decloaking Chamber, Biocare Medical, Concord, USA) at 95°C after deparaffinization. Then, the histological sections were immersed in 3% hydrogen peroxide solution (1h 20 min) and in 1% bovine serum albumin (12 h) to block the endogenous peroxidase activity and nonspecific sites. They were incubated with one of the primary antibodies (rabbit primary antibodies): anti-TGF-β1 (orb11468, Biorbyt, San Francisco, USA), anti-FGF-2 (SC-365106, Santa Cruz Biotechnology, Inc., Heidelberg, BW, Germany), and anti-VEGF (orb191500, Biorbyt),), which were diluted (Antibody Diluent with Background Reducing Components, Dako Laboratories, Carpinteria, USA) and placed in a moist chamber (24 h). The histological sections were then incubated with a biotinylated secondary antibody and treated with streptavidin-horseradish peroxidase conjugate (1h 30 min each) (both the biotinylated secondary antibody and streptavidin were from the Universal Dako Labelled HRP Streptavidin-Biotin kit, Dako Laboratories). The reaction was then developed using the chromogen 3,3′-diaminobenzidine tetrahydrochloride (DAB Chromogen kit, Dako Laboratories) and counterstained with hematoxylin. The negative controls were subjected to the procedures without the primary antibodies.

The immunolabeling was evaluated (400× magnification) by a single experienced and calibrated operator blinded to the experimental groups. The root tip and in the center of the apical third of the root canal space were evaluated.^7^ Immunolabeling was defined as the presence of a brownish color in the cytoplasm of the cells. A semi-quantitative analysis was performed in the chosen areas by a unique and blinded examiner, according to number of immunolabeled cells:^27, 28^ 0, immunolabeling missing (absence of labeling in extracellular matrix and complete absence of immunoreactive cells); 1, low pattern of immunolabeling (weak labeling of the extracellular matrix and approximately one-quarter of the immunoreactive cells); 2, moderate pattern of immunolabeling (moderate labeling of the extracellular matrix and approximately one-half of the immunoreactive cells); 3, strong pattern of immunolabeling (strong labeling of the extracellular matrix and approximately three-quarters of the immunoreactive cells); and 4, very strong pattern of immunolabeling (extremely strong labeling of the extracellular matrix and approximately all immunoreactive cells).

### Statistical analysis

Statistical analyses were conducted using SigmaPlot (version 12.0, Systat Software, Inc.) software. Given the absence of normality after Shapiro-Wilk test, the histomorphometry data and the immunolabeling data were analyzed using the Mann-Whitney U-test, comparing experimental groups at a significance level of 5% (p < 0.05).

## Results

### Cementum-like tissue analysis

**Figure 1:**
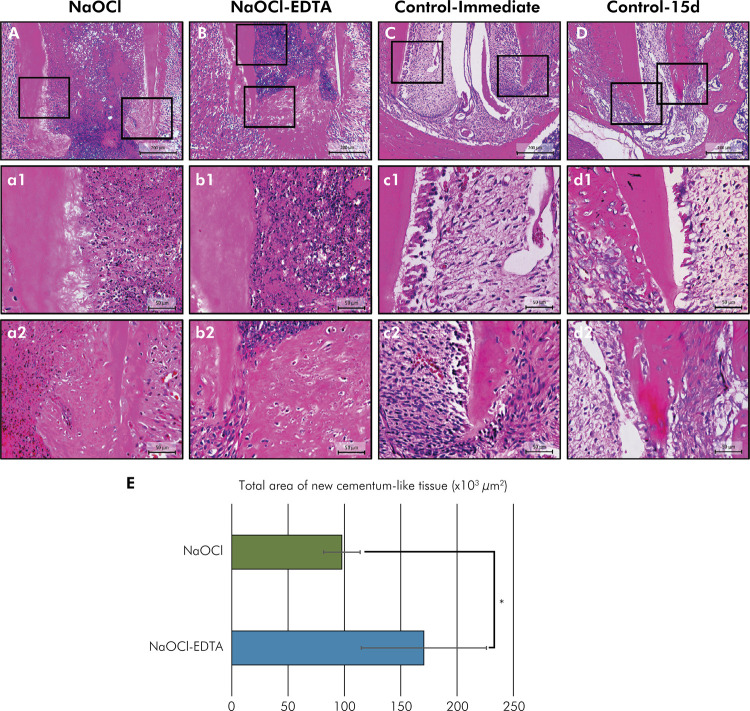
Representative images of histological analysis. (A-a2, B-b2) NaOCl and NaOCl-EDTA: (A, B) panoramic microscopic aspect of the apical third with new cememtum-like tissue, (a1, b1) apical third with inflammatory cells and blood clot, (a2, b2) root tip area of new cementum-like tissue, and ingrowth of fibroblast-like cells. (C-c2, D-d2) Control-immediate and Control-15d: (C, D) panoramic microscopic aspect of the apical third showing normal pulp tissue, (C) open apex, and (D) continued root development, (c1-c2, d1-d2) the presence of organized tissue, and normal dentin-pulp complex. (e1-h1, e2-h2). (E) Data of histomorphometric analysis of new cementum-like tissue in the apical area at 15 d. The symbol * indicates significant difference between the experimental groups (p < 0.05). [100×, 400×: Hematoxylin-eosin].

Representative images and data from the histomorphometric assessment are presented in Figure 1 and Table 1. A significant increase in the area of newly formed cementum-like tissue was observed in the dentinal wall around the apical region of the mesial root in the NaOCl-EDTA group compared to the NaOCl group (p < 0.05). In both experimental groups, cells resembling cementoblasts and cementocytes were evident in the new mineralized tissue. In contrast, specimens from the Control-immediate group exhibited organized pulp tissue with open apices, while the Control-15d specimens showed continued root development with progressive apical closure and a typical dentin-pulp complex.

**Table 1:** Area (µm2 ×103) of newly formed cementum-like tissue in the apical third according to the experimental groups.

Groups	Area [median (IQR: Q1; Q3)*]
NaOCl	97.70 (91.35; 107.21)
NaOCl-EDTA	170.52 (139.69; 205.45)
p-value**	= 0.041

*IQR = interquartile range; Q1 = lower quartile (25%); Q3 = upper quartile (75%); **There was significant difference between groups regarding the area of newly formed cementum-like tissue at 15 days (p < 0.05).

### Analysis for the presence of growth factors

**Figure 2:**
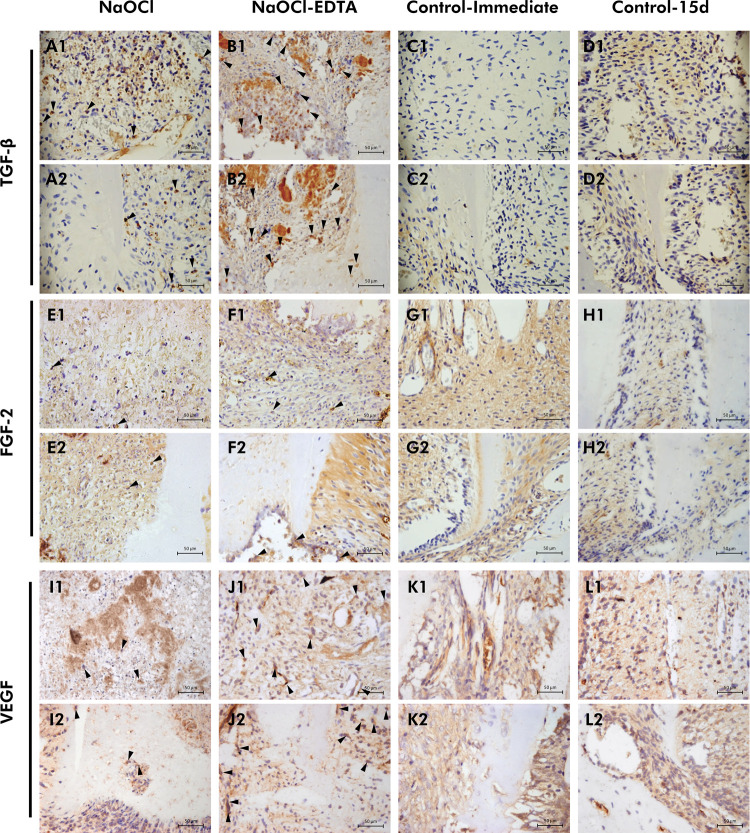
Representative images of TGF-β1, FGF-2, and VEGF immunolabeling. (a1-d1, a2-d2) TGF-β1: (a1, a2) NaOCl shows low-to-moderate immunolabeling, and (b1, b2) NaOCl-EDTA shows moderate-to-very strong immunolabeling. (e1-h1, e2-h2) FGF-2: (e1, e2) NaOCl and (f1, f2) NaOCl-EDTA shows low-to-moderate immunolabeling. (i1-l1, i2-l2) VEGF: (i1, i2) NaOCl shows moderate immunolabeling, and (j1, j2) NaOCl-EDTA shows strong-to-very strong immunolabeling. Black arrowheads indicate immunolabeled cells. [400×: Immunolabeling for TGF-β1, FGF-2, and VEGF].

Representative images and data from the TGF-β1, FGF-2, and VEGF immunolabeling analyses are presented in Figure 2 and Table 2. Over the 15-day period, the NaOCl-EDTA group significantly differed from the NaOCl group (p < 0.05), exhibiting very strong TGF-β1 immunolabeling in the root tip region and strong immunolabeling in the apical third of the mesial root canal space. In contrast, the NaOCl group showed low-to-moderate immunolabeling in both areas. For FGF-2, both experimental groups exhibited a similar immunolabeling in the evaluated regions, with low-to-moderate immunolabeling in the root tip area and predominantly moderate immunolabeling in the apical third (p > 0.05). Regarding VEGF immunolabeling, the NaOCl-EDTA group showed a significant difference compared to the NaOCl group (p < 0.05), presenting strong to very strong immunolabeling in the root tip and apical third of the root canal space, respectively. In contrast, the NaOCl group displayed moderate immunolabeling in both locations.

**Table 2:** Scores for the immunolabeling of TGF-β1, FGF-2, and VEGF in each location of groups.

Location	Scores	TGF-β1		FGF-2		VEGF	
		NaOCl	NaOCl-EDTA	NaOCl	NaOCl-EDTA	NaOCl	NaOCl-EDTA
Root tip	0	0/6	0/6	0/6	0/6	1/6	0/6
	1	3/6	0/6	2/6	2/6	3/6	0/6
	2	3/6	2/6	3/6	2/6	2/6	4/6
	3	0/6	1/6	1/6	2/6	0/6	2/6
	4	0/6	3/6	0/6	0/6	0/6	0/6
	Median*	1.5^a^	3.5^b^	2^a^	2^a^	2^a^	3^b^
	p-value	= 0.015		= 0.818		= 0.026	
Apical third of pulp	0	0/6	0/6	0/6	0/6	1/6	0/6
	1	3/6	0/6	2/6	1/6	4/6	1/6
	2	2/6	1/6	2/6	2/6	1/6	2/6
	3	1/6	4/6	1/6	2/6	0/6	1/6
	4	0/6	1/6	1/6	1/6	0/6	2/6
	Median*	1.5^a^	3^b^	2^b^	2.5^a^	2^a^	3.5^b^
	p-value	= 0.026		= 0.589		= 0.026	

*Different letters on the same line indicate statistically significant differences between groups for each marker at each location (p < 0.05).

## Discussion

This study investigated the influence of EDTA irrigation on the formation of cementum-like tissue and the presence of growth factors (TGF-β1, FGF-2, and VEGF) following REPs in an animal model. The results showed that final irrigation with 17% EDTA significantly increased the area of the newly formed cementum-like tissue and enhanced TGF-β1 and VEGF immunolabeling in the root tip and apical third of the root canal space in immature rat molars. Conversely, FGF-2 immunolabeling was unaffected. Therefore, the null hypotheses of the study were only partially accepted.

During REP, immature teeth undergo chemical treatment with irrigating solutions to facilitate the removal of microorganisms and remnants of necrotic tissue.^29^ Additionally, this procedure aims to release signaling molecules from conditioned dentin.^29^ The choice of 2.5% NaOCl and 17% EDTA was based on the protocols described for REPs,^16,17^considering the antibacterial and solvent actions of NaOCl,^18,30^ as well as the chelating potential of EDTA.^4,18,31^

After EDTA irrigation, the released growth factors may diffuse into the blood clot, promoting cell migration and stimulating cell differentiation upon contact with dentin by stem cells.^4,14^ Higher growth factor release is directly correlated with prolonged EDTA exposure.^21^ Several in vitro studies have assessed the effects of EDTA on growth factor release, primarily using dentin discs or dentin extracts.^3,9,11,21,30,32^ However, these evaluations do not replicate the clinical scenario, wherein, post-irrigation, intracanal bleeding is initiated by intentional laceration/puncture of periapical tissues. Furthermore, there were not enough in vivo studies evaluating the influence of EDTA on the release of growth factors during REPs.

Similar to other histologic and radiographic analyses,^20,33,34^ this investigation demonstrated hard tissue deposition following REPs, particularly on the root canal walls. However, this study also revealed a significant increase in the area of newly formed mineralized tissue with EDTA use. This neoformed tissue exhibited characteristics of cementum and likely originated from stem cells of the periodontal ligament or apical papilla. In contrast, a prior study found no influence of EDTA irrigation on mineralized tissue formation in rat molars during REPs.^7^ This disparity could be attributed to differences in the chosen assessment method. Whereas the prior study^7^ employed a scoring system to evaluate newly formed mineralized tissue, the current study used histomorphometry. Thus, these findings suggest that histomorphometric analysis may offer more precise measurements for this parameter.^35^ Another potential factor contributing to the differing results might be the primary objectives of the two investigations. Unlike the previous study focused on complete root development and apical closure, this study aimed to quantify the newly formed mineralized tissue post-REPs. The enhanced hard tissue deposition observed may be linked to the favorable environment fostered by EDTA, promoting the release of dentin matrix-derived growth factors associated with tissue mineralization, such as TGF-β1.^21,29^

TGF-β plays a pivotal role in REPs,^19^ particularly in odontoblastic differentiation and dentinogenesis.^11,29^ This growth factor also appears to mediate the expression of key pro-angiogenic proteins involved in tissue regeneration, such as FGF-2 and VEGF, through a Smad signaling pathways. ^36,37^ These released growth factors may activate the mesenchymal stem cells, which, in turn can be detected by immunoassays. In this study, increased immunolabeling of TGF-β1 was observed in the apical region of roots irrigated with 17% EDTA. These results are consistent with in vitro studies and existing in vivo data using mice molars,^2^ where EDTA irrigation - either alone^2,29^ or in combination with NaOCl^13,22^ - effectively released TGF-β1 from conditioned dentin using various analysis methods.

Furthermore, higher VEGF immunolabeling was observed with EDTA use. Conversely, a systematic review^14^ indicated that EDTA irrigation had no significant impact on the VEGF release in in vitro^19,38,39^or animal studies.^2^ These controversial results might be attributed to differences in conditioning protocols, experimental models, and analysis methods. Additionally, extended observation periods might diminish VEGF expression due to its short half-life and basal levels in dentin.^14,19^ Nonetheless, angiogenesis, mediated by VEGF remains a critical process during tissue regeneration, particularly in the early stages of wound healing.^40^ In this study, the enhanced TGF-β1 and VEGF immunolabeling could be attributed to the dentin matrix solubilization facilitated by EDTA^18^ and the 15-day evaluation period.

However, EDTA did not influence FGF-2 immunolabeling. Previous research demonstrated significant FGF-2 release from human root fragments only when EDTA was combined with adipose-derived mesenchymal stem cells, highlighting the pivotal role of these cells in its secretion.^19^ On the other hand, the quantity and distribution of bioactive molecules released into the root canal may vary over time, suggesting that a significant liberation of these factors could occur over extended periods,^2,14^ warranting further investigation.

Moreover, a limitation of the present study was the lack of investigating into the influence of EDTA during REPs in infected root canals. Persistent inflammation and bacterial infection within the root canal are known to hinder the effect of dentin-released proteins in this microenvironment, potentially compromising tissue regeneration^6,7^. Some studies have reported minimal effects of this solution or low values of TGF-β1 release in the presence of biofilm in extracted root segments,^22,32^ underscoring the need to validate these findings in contaminated models. Additionally, our study was restricted to a 15-day evaluation period, and variations in growth factors distribution could emerge with long-term longitudinal assessments. In summary, this study suggests that EDTA may enhance the deposition of newly formed cementum-like tissue, potentially strengthening the root walls of immature teeth. The increased presence of TGF-β1 and VEGF may contribute to improved hard tissue deposition.

## Conclusion

EDTA irrigation during REPs increased the formation of newly formed cementum-like tissue, as well as the presence of TGF-β1 and VEGF in the apical area but did not influence FGF-2.

## Data Availability

After publication the data will be available on demand to the authors - condition justified in the manuscript.
